# A systematic review and meta-analysis of low back pain and its associated factors among school teachers in Africa

**DOI:** 10.1186/s12891-023-06633-1

**Published:** 2023-06-17

**Authors:** Amensisa Hailu Tesfaye, Giziew Abere, Tesfaye Hambisa Mekonnen, Abdisa Gemedi Jara, Fantu Mamo Aragaw

**Affiliations:** 1 Department of Environmental and Occupational Health and Safety, Institute of Public Health, College of Medicine and Health Sciences, University of Gondar, P.O. Box 196, Gondar, Ethiopia; 2 Department of Clinical Pharmacy, School of Pharmacy, College of Medicine and Health Science, University of Gondar, P.O. Box 196, Gondar, Ethiopia; 3 Department of Epidemiology and Biostatistics, Institute of Public Health, College of Medicine and Health Sciences, University of Gondar, P.O. Box 196, Gondar, Ethiopia

**Keywords:** Low back pain, Musculoskeletal pain, Risk factors, School teachers, Africa

## Abstract

**Introduction:**

Low back pain (LBP) is a key social, economic, and public health problem in the world. The impact of LBP is given less priority and is empirically unrepresented in low- and middle-income countries as a result of the focus on more pressing and life-threatening health issues, including infectious diseases. In Africa, the prevalence of LBP is irregular and increasing among schoolteachers on account of teaching activities performed under suboptimal working conditions. Therefore, the objective of this review was to estimate the pooled prevalence and associated factors of LBP among school teachers in Africa.

**Methods:**

This systematic review and meta-analysis was designed based on the PRISMA guidelines. A comprehensive systematic literature search focused on LBP in African school teachers was conducted using the PubMed/MEDLINE, CINAHL, and CABI databases, regardless of publication timelines, from October 20 to December 3, 2022. In addition, gray literature was searched using Google Scholar and Google Search. Data were extracted in Microsoft Excel by using the JBI data extraction checklist. The overall effect of LBP was estimated using a random effect model via DerSimonian-Laird weights. The pooled prevalence and odds ratio of associated factors with 95% CI were computed using STATA 14/SE software. The I^2^ test and Egger’s regression test were used to assess heterogeneity and publication bias, respectively.

**Results:**

A total of 585 articles were retrieved, and 11 eligible studies involving a total of 5,805 school teachers were included in this systematic review and meta-analysis. The overall estimated pooled prevalence of LBP in African school teachers was found to be 59.0% (95% CI: 52.0%–65.0%). Being female [POR: 1.53; 95% CI (1.19, 1.98)], being older [POR: 1.58; 95% CI (1.04, 2.40)], being physically inactive [POR: 1.92; 95% CI (1.04, 3.52)], having sleep problems [POR: 2.03; 95% CI (1.19, 3.44)] and having a history of injury [POR: 1.92; 95% CI (1.67, 2.21)] were factors significantly associated with LBP.

**Conclusions:**

The pooled prevalence of LBP was high among school teachers in Africa compared to developed nations. Sex (female), older age, physical inactivity, sleep problems, and a history of previous injury were predictors of LBP. It is suggested that policymakers and administrators ought to gain awareness of LBP and its risk factors to put existing LBP preventive and control measures into action. Prophylactic management and therapeutic strategies for people with LBP should also be endorsed.

**Supplementary Information:**

The online version contains supplementary material available at 10.1186/s12891-023-06633-1.

## Introduction

Low back pain (LBP) is defined as pain or discomfort from the region of the twelfth (12^th^) rib to the region of the inferior gluteal folds, with or without accompanying leg pain [1]. LBP is the most widespread musculoskeletal condition as well as the leading cause of years lived with disability (YLDs) worldwide [2, 3]. As part of the Global Burden of Disease (GBD) study expert group, LBP is among the top ten high-burden diseases and injuries, with an average number of disability-adjusted life years (DALYs) higher than HIV, road injuries, tuberculosis, lung cancer, chronic obstructive pulmonary disease, and preterm birth complications [4]. As a result, the condition is a significant public health concern because it has a noticeable impact on healthcare systems and people’s quality of life (QoL) [5]. In contemporary workplaces, the occupational application of LBP is strong-minded in terms of direct and indirect costs, including lost productivity, activity limitation, time away from work, early retirement, and incurring a major economic burden in compensation costs and lost wages [6–8].

Low back pain is a major clinical and governmental health problem that has reached epidemic levels. It is estimated that about 90% of the global population suffers from LBP at some point in their lives [9]. LBP was identified by the WHO as one of the top three occupational health problems, and it has been estimated that occupational exposures accounted for 37% of the global burden of LBP [10, 11]. The prevalence of LBP is increasing globally at an estimated 30% to 80% among the general population [12]. It varies according to the type of activity performed [13–16] and affects adults of all ages, any gender, and any socioeconomic background [17].

School teachers are among the occupational groups most affected by LBP [14]. The prevalence of LBP is much higher in teaching staff compared to other occupational groups, ranging from 12 to 95% [18, 19]. The burden of LBP is greater in low- and middle-income countries (LMICs) like African nations [20, 21]. A sparse study conducted among teachers in Africa showed that the prevalence of LBP among teachers is irregular and increasing. In light of this, the prevalence of LBP among school teachers in Africa ranges from 53.2% in Egypt [22], 55.7% in Botswana [16], 62.7% in Nigeria [23], 64.98% in Kenya [24], 74.8% in Ethiopia [25], to 82.9% in Tanzania [18].

Several factors have been associated with the development of LBP in school teachers [26]. The work of a teacher is planning, organizing, and implementing an instructional program in a learning environment to guide students in developing and achieving their academic potential. The majority of a teacher's time is spent standing in the classroom, walking while teaching, writing on the blackboard, preparing lessons, grading assignments, and doing other administrative tasks with risky working postures for extended periods, all of which stress the musculoskeletal system and development of LBP [27, 28]. In Africa, almost all teaching activities are performed under unfavorable conditions, requiring teachers to use their physical, cognitive, and effective capabilities to achieve a teaching objective while over-demanding or generating effort for their psycho-physiological functions [29]. Psychosocial factors such as high workload, sleep disturbance, low job satisfaction, and job stress have been implicated as risk factors for LBP among school teachers [25, 30–32], while some factors like regular exercise and good job satisfaction act as protective effects against LBP in teachers [30, 33]. Socio-demographic factors, such as age, sex, work experience, and lifestyle factors, such as smoking and BMI, are other risk factors associated with LBP among school teachers [16, 24, 34, 35].

The impact of LBP is given less priority and is empirically unrepresented in low- and middle-income countries as a result of the focus on more pressing and life-threatening health issues like none communicable diseases and infectious diseases. In addition, most African countries are in the midst of socio-political crises, and as a result, almost all of each country's resources are diverted to dealing with these political crises rather than addressing public health problems, including the management of LBP [36, 37]. Teachers’ health has a substantial effect on a country's development since they are valuable resources for the socio-economic and cultural development of that country. As a result, an increase in the prevalence of LBP among teachers will affect every country's education system. In Africa, the prevalence of LBP is scattered and increasing in the teaching profession; however, there is a lack of studies to estimate the cumulative prevalence and associated risk factors of LBP among school teachers to provide strong evidence for policymakers and implementers to design effective prevention and control methods to protect teachers’ health and the quality of education that their students receive. Therefore, the objective of this review was to estimate the pooled prevalence and associated factors of LBP among school teachers in Africa. The review focused on nursery, primary or elementary, secondary, and preparatory school teachers.

## Materials and methods

### Registration and protocol

This systematic review and meta-analysis were done to compile the most recent evidence using published and gray literature on the prevalence and associated risk factors of LBP among school teachers in Africa. The protocol for this review was registered on the Prospective Register of Systematic Reviews (PROSPERO) international database (protocol registration number: CRD42022373366). For reporting, we followed the protocol of the Preferred Reporting Items for Systematic Review and Meta-Analysis (PRISMA) guidelines [38] (S1 File**)**.

## General characteristics of the reviewed articles

All studies primarily reporting on the prevalence of LBP among school teachers in African countries are included in the current review. As well, studies reported on the prevalence of MSDs as a whole, but they had to provide subgroup data for LBP prevalence, which could also be included in our systematic and meta-analytical review. Subjects in the studies could be of any race, gender, and people the age of 18 years and above.

## Search strategies

A comprehensive systematic literature search was conducted using the PubMed/MEDLINE, CINAHL, and CABI databases, regardless of publication timelines, from October 20 to December 03, 2022, because there had been no systematic review and meta-analysis done before. The following key terms were used in combination with the Boolean operators "AND" and "OR". For the PubMed/MEDLINE search, the following key terms were used in combination with the Boolean operators "AND" and "OR". ((((((((((((((low back pain*[All Fields]) OR (lower back pain*[All Fields])) OR (back pain*[All Fields])) OR (back ache*[All Fields])) OR (Low Backache*[All Fields])) OR (Back Ache*[All Fields])) AND (Primary school teacher*[All Fields])) OR (Elementary school teacher*[All Fields])) OR (Middle School Teacher*[All Fields])) OR (Pre-School Teacher*[All Fields])) OR (Secondary school teacher*[All Fields])) OR (Preparatory school teacher*[All Fields])) OR (School teacher*[All Fields])))) OR (High school teacher*[All Fields]) AND (Algeria OR Angola OR Benin OR Botswana OR Burkina Faso OR Burundi OR Cameroon OR Cape Verde OR Central African Republic OR Chad OR Comoros OR Congo OR Ivory Coast OR Djibouti OR Egypt OR Equatorial Guinea OR Eritrea OR Ethiopia OR Gabon OR Gambia OR Ghana OR Guinea OR Guinea-Bissau OR Kenya OR Lesotho OR Liberia OR Libya OR Madagascar OR Malawi OR Mali OR Mauritania OR Mauritius OR Morocco OR Mozambique OR Namibia OR Niger OR Nigeria OR Rwanda OR Sao Tome and Principe OR Senegal OR Seychelles OR Sierra Leone OR Somalia OR South Africa OR Sudan OR Swaziland OR Tanzania OR Togo OR Tunisia OR Uganda OR Zambia OR and Zimbabwe). In addition to the electronic database search, gray literature was searched using direct Google Search and Google Scholar. Reference lists (bibliographies) of the included studies were also searched to obtain additional articles.

## Eligibility criteria

### Inclusion criteria

Articles that met the following determined inclusion criteria were included in this systematic review and meta-analysis: (a) were published and unpublished studies in any period (the study period was not restricted for inclusion), (b) examined the prevalence and/or associated factors of LBP, (c) were conducted in African countries, (d) studies that employed an observational study design, (e) were carried out on primary school teachers, elementary school teachers, middle school teachers, pre-school teachers, secondary school teachers, and/or preparatory school teachers, and (f) studies reported in the English language up to December 03, 2022, were included in our systematic review and meta-analysis.

## Exclusion criteria

Qualitative studies, systematic reviews, editorial letters and reviews, short communications, and commentaries were excluded. In addition, articles that were not fully accessible after three or more personal email contacts with the corresponding author were also excluded.

## Study selection

Two review authors (AHT & FMA) independently screened articles by their title, abstract, and full text to identify eligible articles using predetermined inclusion and exclusion criteria. The identified articles were then combined, exported, and managed using Endnote X9.2 (Thomson Reuters, Philadelphia, PA, USA) software. After the exclusion of duplicate studies, titles, and abstracts were independently screened for inclusion in the full-text appraisal, which was done by other review authors (AHT, GA, & FMA), and the disagreement between authors that arises during data abstraction and selection are solved based on evidence-based discussion and by the involvement of the remaining review authors (THM & AGJ).

## Outcome measurements

The primary outcome of this systematic review and meta-analysis was the prevalence of LBP among school teachers in Africa, which indicates the number of people who reported the pain divided by the population number at a given point in time (in our case, 12 months) and presented as a prevalence or proportion. We used the authors’ reported definitions using standardized scales or questionnaires (e.g., standardized Nordic questionnaire, Dutch musculoskeletal questionnaire). The secondary outcomes of this review were to identify the risk factors for LBP (e.g., sociodemographic factors like sex, age, etc., personal or behavioral factors like physical exercise, BMI, smoking, etc., psychosocial factors, ergonomics factors, and workplace-related factors). We derived estimates from the number of cases and sample size provided in each study when the outcome of the results (e.g., proportion and 95% CI) were not directly reported and it was practical to do so. The measure of effect size was the odds ratio.

## Data extraction and management

The data were extracted using the Joanna Briggs Institute (JBI) data extraction checklist. Three review authors (AHT, FMA, and GA) extracted the data independently using a Microsoft Excel spreadsheet. The differences between the three review authors were solved with discussion. Any discrepancies were resolved through a review by the other review authors (THM and AGJ). From each study, information such as the name of the first author, the year of publication, the study year, the study country, the study design, the total number of participants, the number of LBP cases among school teachers, the prevalence of LBP and its standard error, factors associated with LBP with their standard errors, and each study's measures of association were extracted.

## Assessment of the methodological quality of the studies

The JBI quality appraisal tool for cross-sectional studies was used to assess the quality of included articles and the risk of bias in each study [39]. Three reviewer authors (AHT, GA, and FMA) independently assessed the quality of the included articles. The assessment tool contains eight criteria: (1) clear inclusion and exclusion criteria; (2) description of the study subject and study setting; (3) use of a valid and reliable method to measure the exposure; (4) standard criteria used for measurement of the condition; (5) identification of confounding factors; (6) development of strategies to deal with confounding factors; (7) use of a valid and reliable method to measure the outcomes; and (8) use of appropriate statistical analysis. It was evaluated using the JBI critical appraisal checklist options of "yes," "no," "unclear," and "not applicable." The risks for biases were classified as low (total score, 5 to 8) and high (total score, 0 to 4). The study scored 50% or higher on all quality-assessed items, which were considered low-risk and included in this review. Disagreements that arose during the full-text quality assessment were resolved through evidence-based discussion with the other review authors (THM and AGJ).

## Data synthesis and analysis

The extracted data were exported from a Microsoft Excel spreadsheet to STATA version 14 for further analysis. We calculated the I^2^ statistic to determine study heterogeneity, which describes the percentage of total variation among studies that is due to heterogeneity rather than chance, in which 25%, 50%, and 75% represented low, moderate, and high heterogeneity, respectively [40]. The test statistic showed there is significant heterogeneity among the studies (I^2^ = 96.27%, p < 0.000). Therefore, the overall effect of LBP among African school teachers was estimated using a random effect model and measured by prevalence rates and odds ratios with 95% CI using DerSimonian-Laird weight [41]. We conducted a country-specific sub-group analysis. Furthermore, sensitivity analysis is performed to determine the impact of individual studies on the pooled estimate. Publication bias (the small study effect) was detected using the visual funnel plot test and Egger’s statistical test [42]. To estimate the relationship between LBP and factors, the odds ratio with a 95% confidence level was used, and a significance level of 0.05 was considered for the *P*-value. The results were presented using graphs, tables, texts, and a forest plot.

## Results

### Searching results

**Fig. 1:**
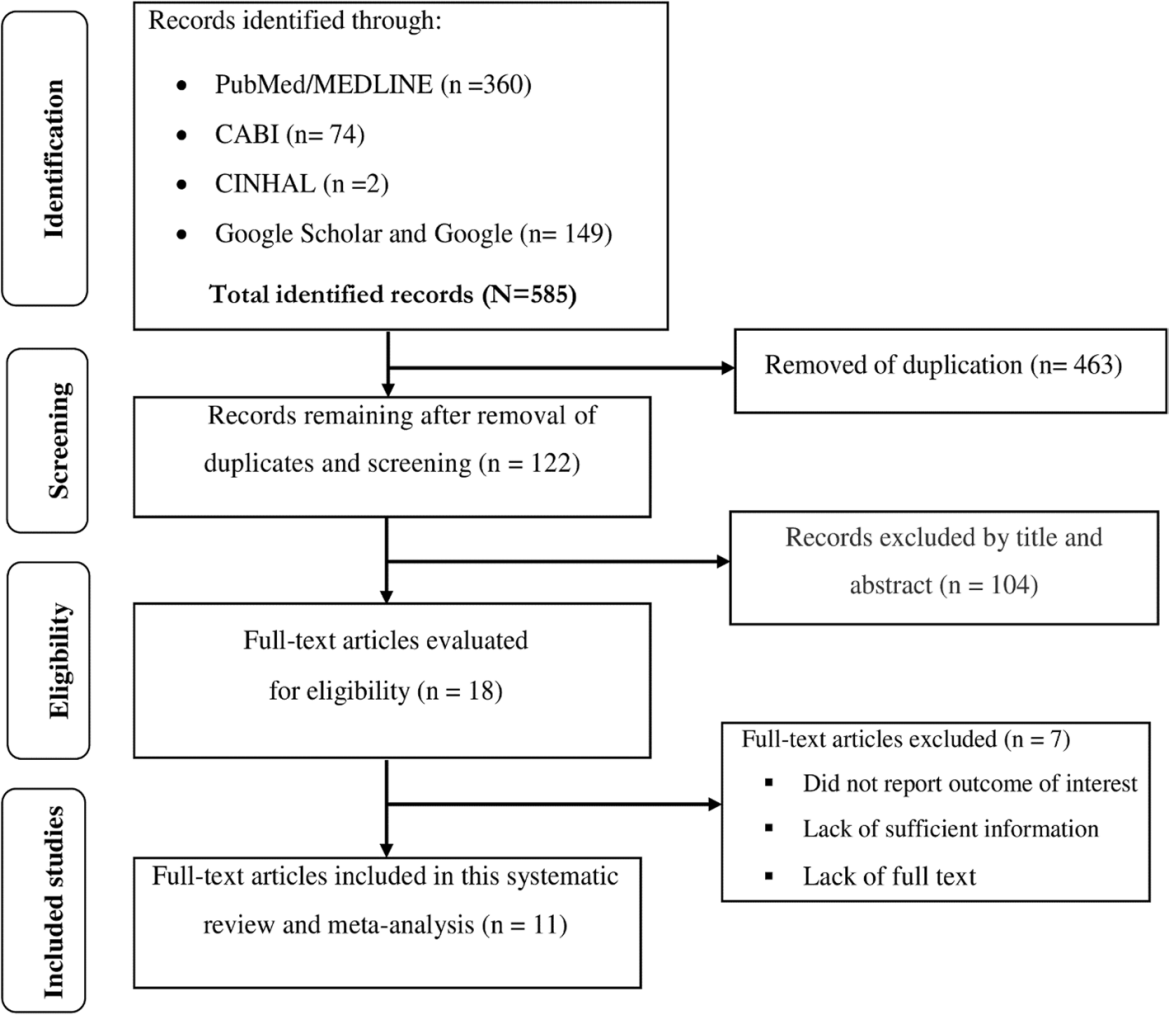
PRISMA flow diagram describing the selection of studies for the systematic review and meta-analysis of low back pain and associated risk factors among school teachers in Africa, 2022

A total of 585 articles were retrieved from PubMed/MEDLINE, CINAHL, and CABI electronic databases, as well as from Google Scholar and Google Search. 463 duplicate articles were removed using EndNote citation manager; 104 articles were excluded after the title and abstract screening, and then 18 articles remained. After a careful inspection of 18 publications for the presence of the outcome variable, 7 papers were removed in compliance with the exclusion criteria, and finally, 11 articles remained. Accordingly, 11 eligible studies were incorporated for this systematic review and meta-analysis to estimate the pooled prevalence of LBP and its associated factors after quality assessment using the JBI quality assessment critical appraisal checklist. The overall study selection process was represented by the following flow diagram (Fig. 1).

## Description of included studies

The characteristics of the review, which include the publication year, sample size, study design, and study country, are all compiled in Table 1. All included studies were cross-sectional in study design, with sample sizes ranging from 153 in Nigeria [23] to 1747 in Botswana [16]. The studies were published from 2013 through 2022. The entire set of included studies counted for both genders and the recall periods for LBP were reported as a 12-month prevalence.

**Table 1:** A descriptive summary of the included articles for the systematic review and meta-analysis of the prevalence and associated factors of LBP among school teachers in Africa, 2022

Author (s) and Publication Year	Country	Population	Study design	Sample size	Response rate	Prevalence (%)	Quality status
T Gebreyesus et al., 2019 [43]	Ethiopia	Kindergartens, elementary and secondary school teachers	CS	406	96.4%	54.90	Low risk
Abebaw T-A et al., 2018 [44]	Ethiopia	Primary School teachers	CS	771	93.2%	44.00	Low risk
Kebede A et al., 2019 [25]	Ethiopia	Primary School teachers	CS	611	93%	74.80	Low risk
Beyen TK et al., 2013 [30]	Ethiopia	Primary, secondary school, and, senior school teachers	CS	602	90.9%	57.50	Low risk
Mwangi A et al., 2019 [24]	Kenya	primary school teachers	CS	417	90.7%	64.98	Low risk
Ndonye NA et al., 2019 [45]	Kenya	primary school teachers	CS	302	78.6%	58.60	Low risk
Fahmy VF et al., 2022 [22]	Egypt	Primary, secondary, and preparatory school teachers	CS	310	85%	53.20	Low risk
Mo E, Ebied E et al., 2015 [46]	Egypt	Primary School Teachers	CS	200	80%	41.00	Low risk
Erick PN et al., 2014 [16]	Botswana	Primary and secondary school teachers	CS	1747	56.3%	55.70	Low risk
Sumaila FG et al., 2015 [23]	Nigeria	Secondary school teachers	CS	153	76.5%	54.20	Low risk
Nilahi CD et al., 2014 [18]	Tanzania	Primary School Teachers	CS	286	77.5%	82.90	Low risk

*CS* Cross-sectional, Low risk: a study scored ≥ 50% in the JBI quality assessment scale

In this systematic review and meta-analysis, a total of 5,805 school teachers were included to estimate the pooled prevalence of LBP. The eleven studies were carried out across several African nations: Four (36.36%) articles [25, 30, 43, 44] were conducted in Ethiopia, two (18.18%) articles [24, 45] were conducted in Kenya; two (18.18%) articles [22, 46] were conducted in Egypt; and one (9.1%) article was conducted in each of Botswana [16], Nigeria [23], and Tanzania [18]. The lowest and highest prevalence of LBP among school teachers in Africa was 41% in Egypt [46] and 82.9% in Tanzania [18], respectively. All of the articles were re-evaluated by independent evaluators prior to any analysis, and the studies were fit in terms of quality (quality score ranged from 5 to above points). The description of the included studies is presented in Table 1.

### Meta-analysis

## Pooled prevalence of low back pain among school teachers in Africa

**Fig. 2:**
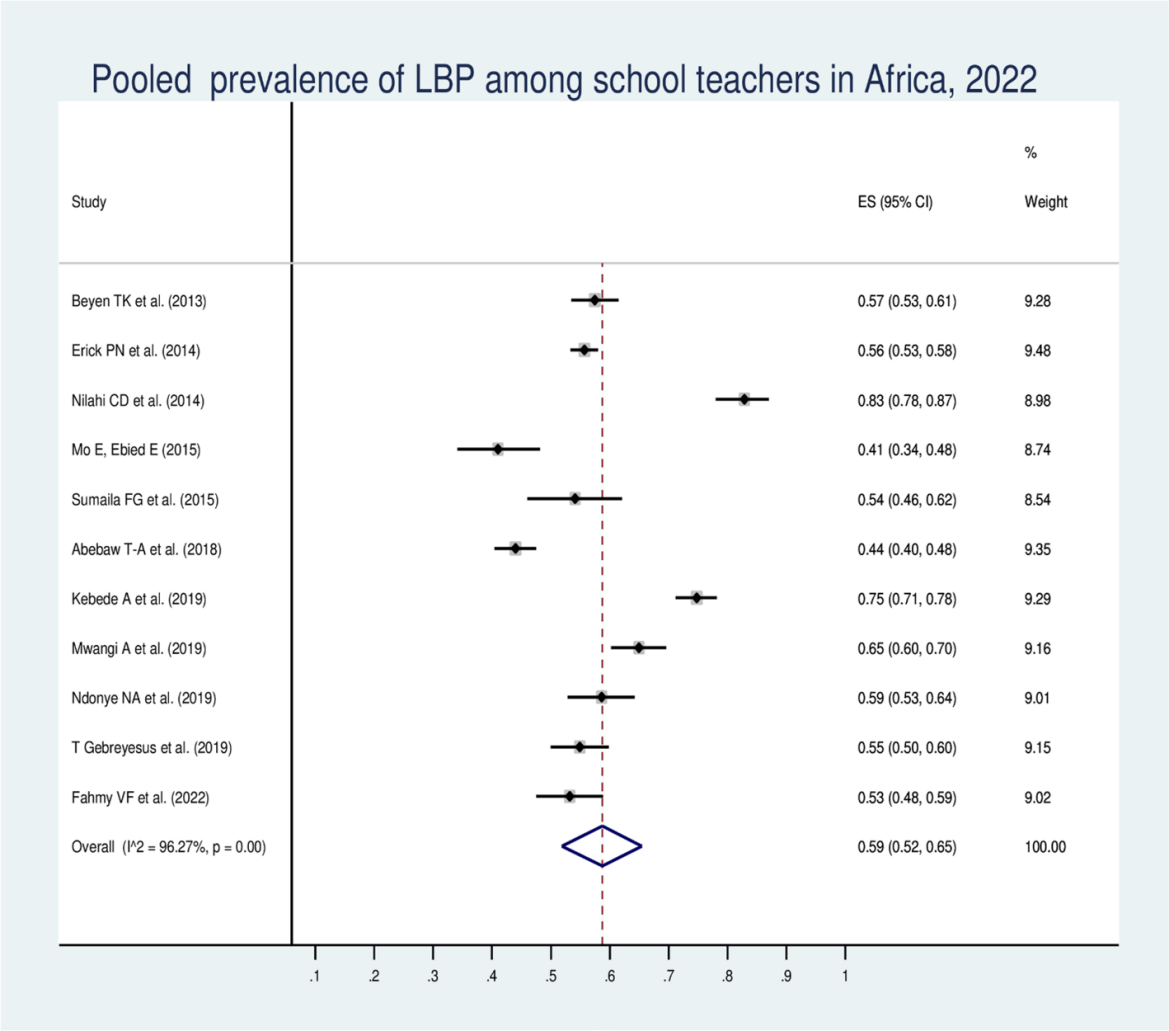
Forest plot of the pooled prevalence of LBP among African school teachers, 2022

There were a total of 5,805 school teachers included in the eleven studies that were used to estimate the pooled prevalence of LBP for the current study. The forest plot result of the eleven included studies showed that the overall pooled prevalence of LBP among African school teachers was 59.0% (95% CI: 52.0–65.0%) (Fig. 2). In addition, the forest plot of our meta-analysis showed that the highest proportion (83.0%) of LBP was reported from a study done in Tanzania [18]. Whereas, the lowest proportion (41.0%) of LBP was reported in a study done in Egypt [46]. Nine (81.82%) of the eleven studies revealed that LBP affected more than 50% of school teachers. According to this review's I2 test statistics (I2 = 96.27%), the included studies showed high heterogeneity. Therefore, subgroup and univariate meta-regression analyses were conducted to identify the possible sources of heterogeneity.

### Subgroup analysis

**Fig. 3:**
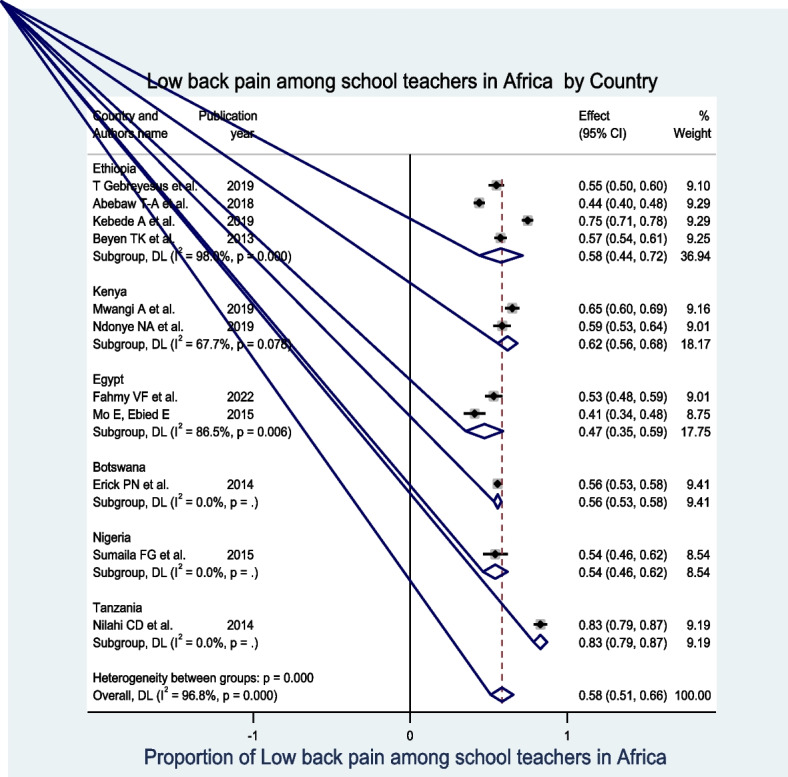
Forest plot of pooled low back pain among school teachers in Africa by country, 2022

The subgroup prevalence of LBP was estimated by considering the country where the studies were conducted. Accordingly, in the subgroup analysis, the overall pooled prevalence of LBP based on the country of the studies was 58.0% (95% CI: 51.0%, 66.0%). In addition, the pooled prevalence of LBP was widely varied across the country. As such, in the country subgroup analysis, Kenya had the highest pooled proportion of LBP at 62.0% (95% CI: 56.0%, 68.0%), followed by Ethiopia at 58.0% (95% CI: 44.0%, 72.0%), and Egypt at 47.0% (95% CI: 35.0%, 59.0%). Even if a single study was reported in Botswana, Nigeria, and Tanzania, the highest prevalence of LBP is reported in a study conducted in Tanzania out of all eleven studies included in this systematic review and meta-analysis (Fig. 3).

## Heterogeneity and small study effect test

**Fig. 4:**
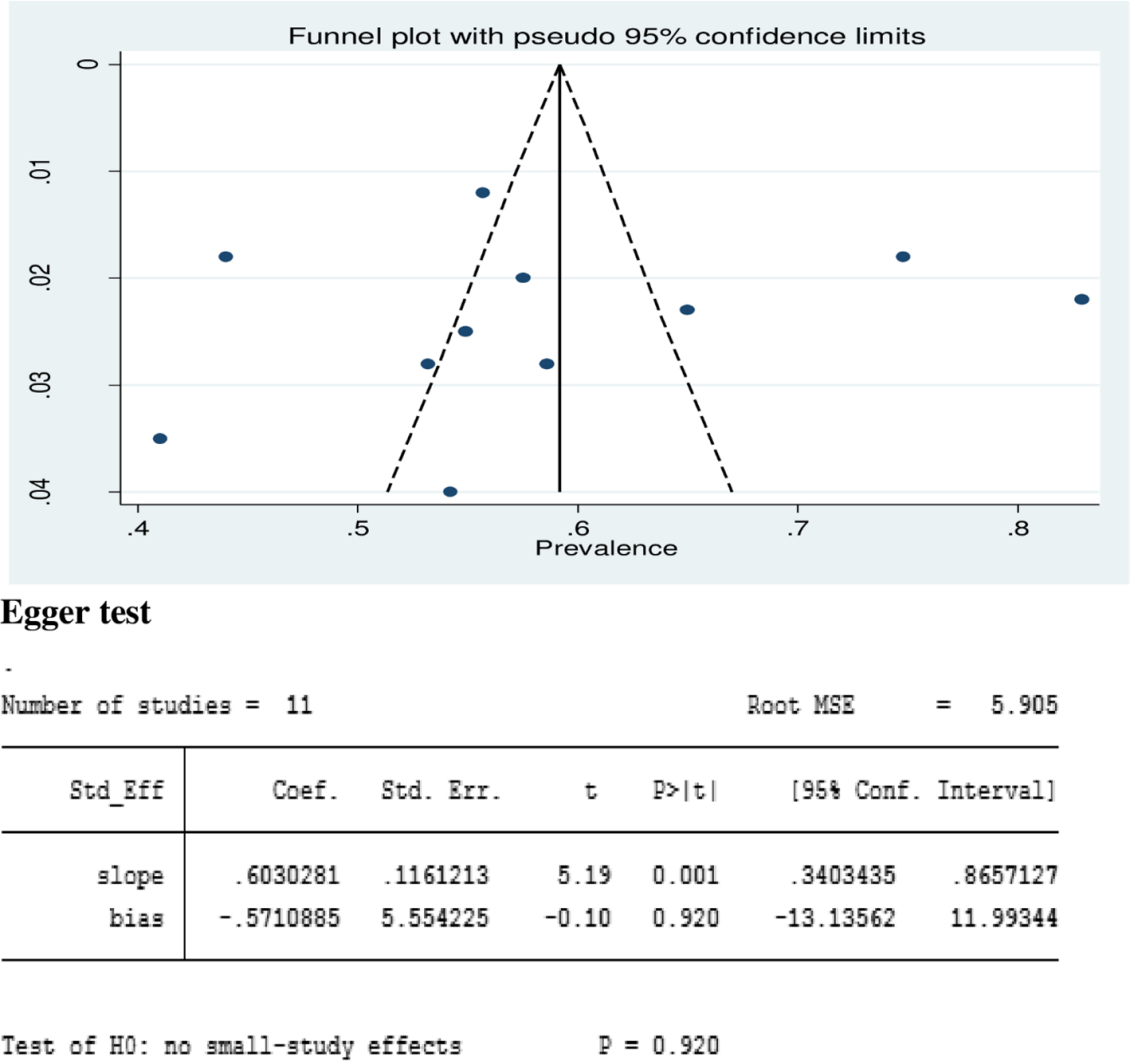
Funnel plot and Egger test of the eleven studies included in the meta-analysis of low back pain among school teachers in Africa, 2022

**Fig. 5:**
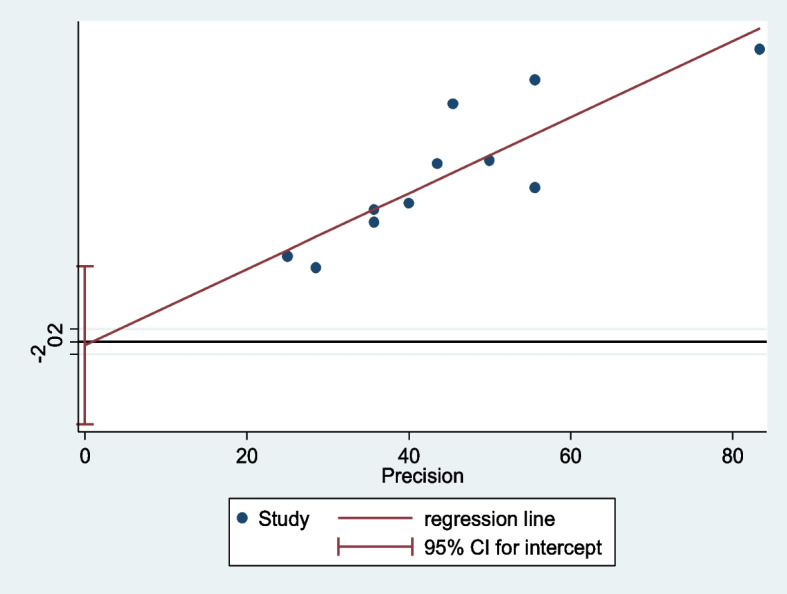
Egger graph to test for small study effect

All eleven included articles were assessed for heterogeneity and publication bias (small study effect). The presence of a possible small study effect was checked by using a funnel plot and the Egger test. Accordingly, the funnel plot (Fig. 4) showed an asymmetric distribution and presented evidence of heterogeneity. However, the result of the Egger regression test indicates that there is no evidence of publication bias (*P*-value = 0.920) (Fig. 5).

## Sensitivity analysis test

**Fig. 6:**
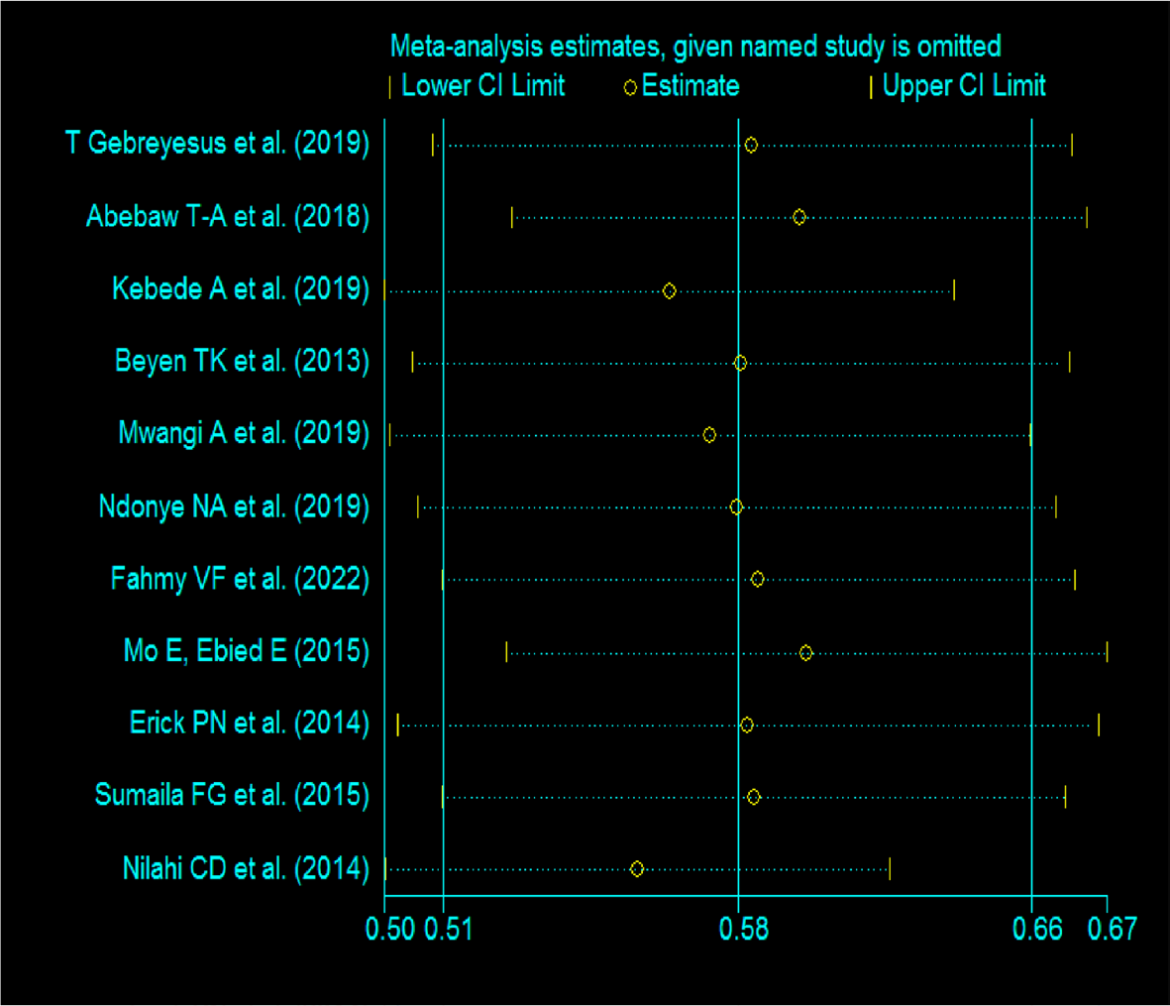
Sensitivity analysis graph to examine the effect of each study on the pooled result

A sensitivity analysis of the eleven studies was conducted to test the effect of each study on the pooled result. This was carried out by excluding each study step by step (i.e., based on eleven studies). However, the results of the sensitivity analysis showed that there were no significant changes across all studies in the fitted meta-analytic model, as shown in Fig. 6.

### Factors associated with low back pain among school teachers in Africa

Factors associated with LBP were identified based on the pooled effect of two or more studies. Gender, age, physical activity, cigarette smoking, sleep problems, and a history of previous injury were identified as factors associated with LBP among African school teachers.

The pooled effect of our review analysis showed that female teachers were 1.53 times more likely to experience LBP compared to male teachers [POR: 1.53; 95% CI (1.19, 1.98)]. Similarly, school teachers who were in the age range of 30 or more years were more likely to have LBP compared to those in the age range of less than 30 years [POR: 1.58; 95% CI (1.04, 2.40)]. As well, the pooled effect of our analysis indicated that the likelihood of suffering from LBP was increased twofold among teachers who did not perform the physical exercise as compared to those who did [POR: 1.92; 95% CI (1.04, 3.52)]. Besides, teachers who had sleep problems were two times more likely to develop LBP compared to teachers who did not have sleep problems [POR: 2.03; 95% CI (1.19, 3.44)]. Furthermore, the odds of having LBP were 1.92 times higher among teachers who had a history of injury compared with those who did not [POR: 1.92; 95% CI (1.67, 2.21)]. However, there was no significant pooled effect size observed between cigarette smoking and LBP among school teachers in Africa in our analysis [POR: 2.86; 95% CI (0.24, 33.54)] (Table 2).

**Table 2:** The pooled effect size of factors associated with low back pain among school teachers in Africa, 2022

Variable (Reference)	Number of studies	Sample size	Effect size (POR with 95% CI)	Heterogeneity		
				Q-value	*P*-value	I^2^
**Sex (male)**	5	3943	1.53 (1.19–1.98)	0.10	0.861	0.0%
Age (< 30 years)	4	3526	1.58 (1.04–2.40)	0.81	0.848	0.0%
Physical exercise (yes)	3	1619	1.92 (1.04–3.52)	0.10	0.953	0.0%
Cigarette smoking (no)	2	1008	2.86 (0.24–33.54)	0.03	0.858	0.0%
Sleep problem (no)	3	1619	2.03 (1.19–3.44)	0.73	0.694	0.0%
History of Injury (no)	2	2349	1.92 (1.67–2.21)	0.00	0.976	0.0%

Keys: *POR* pooled odds ratio, *CI* Confidence interval

## Discussion

Low back pain is the most common public health, economic, and social problem. LBP also has an indiscriminate impact on populations all over the world [47]. This first-of-its-kind systematic review and meta-analysis aimed to estimate the pooled prevalence of low back pain and its associated factors among school teachers in African countries. Overall, this review suggests that LBP pain in teachers is likely an under-researched topic but that the teaching profession itself poses a high risk for LBP. According to this meta-analysis, the overall annual pooled prevalence of LBP among school teachers was found to be 59.0% (95% CI: 52.0–65.0%). As well, based on the combined effects of two or more of the studies included in this meta-analysis, significant factors associated with LBP among school teachers in Africa included gender, age, physical activity, sleep problems, and a history of earlier injuries.

The current pooled prevalence of LBP is in line with a couple of systematic reviews and meta-analyses conducted in Ethiopia (54.2%) [48] and 54.05% [49], a systematic review conducted in Nigeria (55.39%) [50], a systematic review and meta-analysis conducted in India (51%) [51], prior working groups in African nations (57%) [52], as well as a study conducted in the United Arab Emirates (64.6%) [53]. However, the pooled prevalence of LBP in the present review is higher than a systematic review conducted in Latin America (31.3%) [54], a study conducted in the United Kingdom (36.1%) [55], and a study conducted in Spain (20%) [12]. Compared to estimates for the global prevalence of LBP, the African working population has a higher annual prevalence of LBP [12, 56, 57]. Our review finding also revealed that the pooled prevalence of LBP (59.0%) in our finding is higher than the global annual prevalence of LBP (38.5%) [56].

A plausible explanation for the higher pooled prevalence of LBP among African school teachers is that LBP is a low priority in impoverished African countries and receives less attention than other diseases because the governments of such countries concentrate on more urgent and life-threatening health issues, such as infectious diseases [58]. The other reason can be due to the fact that the ongoing COVID-19 epidemic, violent wars, and challenges linked to climate change have severely strained the African healthcare system. As a result, health systems in every African nation are overburdened by communicable and noncommunicable diseases, which makes LBP a lower-priority issue in African countries' health development plans [59, 60]. As well, in every African nation, there is an absence of national screening programs and surveillance data on musculoskeletal disorders (MSDs) and/or LBP, which supplants the existing fact of the absence of priority given to every working population in Africa. Furthermore, the high prevalence of LBP among African school teachers can also be attributed to the fact that these specific working groups are accustomed to working in subpar workplace environments with poor ergonomic circumstances, which exacerbate pain in the low back region [16, 61, 62]. Therefore, it is necessary to create and put into effect plans and policies that effectively address the burden of LBP in developing or underdeveloped nations, such as those in Africa [21].

The result of subgroup analysis by country showed that the pooled prevalence of LBP was almost similar across countries in Africa. Thus far, there has been a relatively higher pooled prevalence of LBP found in Kenya (62% with a 95% CI: 56%, 68%), followed by Ethiopia (58% with a 95% CI: 44%, 72%), and the highest prevalence of LBP is reported in a study conducted in Tanzania out of all eleven studies included in this systematic review and meta-analysis study.

### Risk factors of low back pain among school teachers in Africa

Our systematic review and meta-analysis also tried to investigate factors associated with low back pain. Accordingly, female gender, advanced age, physical inactivity, sleeping problems, and a history of previous injury were the identified predictors for LBP among African school teachers.

This meta-analysis of risk factors showed that the odds of LBP among female teachers were 1.53 times higher when compared with male teachers [POR: 1.53; 95% CI (1.19, 1.98)]. This finding is in agreement with studies conducted in Malaysia [63], Brazil [11], China [27], Turkey [64], and Iran [65], which reported that being female in gender had higher odds of LBP than being male among teachers. As opposed to our findings, few studies in Filipino [31] and Slovenia [66] indicated female gender had no significant effect on LBP in teachers. The plausible reason for this gender difference could be that females have a lower threshold for pain than males [67]. As a result, females were more likely to report pain trouble than their counterparts. In addition, females’ lower physical strength, pregnancy, menstruation, and pain related to osteoporosis might be a possible explanation for the incongruity [33]. Moreover, females could carry out more heavy housework than males daily, and evidence [68] suggests that these differences in household task involvement may explain LBP differences between females and males. Furthermore, the physical stress of child-rearing and perimenopausal abdominal weight gain [69] and the physiologically low disk space in females [70] may also contribute to the occurrence of more LBP among females. This suggests that back pain prevention strategies in the workplace should give much emphasis on the female population.

School teachers who were in the age range of 30 or more years were found to be more likely to have LBP compared to those in the age range of less than 30 years. This finding is supported by previous studies conducted in the Philippines [71], Brazil [11], Turkey [72], Slovenia [66], China [73], and America [74]. Besides, the European Agency for Safety and Health at Work [75] highlighted advanced age as one of the most reported individual risk factors for LBP. This could be justified as teachers age; there is a gradual decline in muscle mass, and they lose connective tissue elasticity and undergo a diminishing of cartilage between joints. On the other hand, healing will slow down with advancing age [76]. In addition, with increasing age, the direct blood supply to the intervertebral disc will be reduced [77]. This will make lumbar discs easily susceptible to wear and tear or degeneration. Lumbar disc degeneration leads to lumbar spine instability. This unstable lumbar spine then imposes a higher biomechanical demand on the ligaments, capsules, muscles, and facets [78] and consequently develops pain in the lower back region. The older teachers might have a longer teaching service, and this increases the duration of exposure to risk factors. As a result, this cumulative effect can also explain the higher odds of LBP among older teachers than their younger colleagues [79].

As well, the pooled effect of this study indicated that the likelihood of experiencing LBP was increased by twofold among school teachers who did not perform the physical exercise as compared to those who did [POR: 1.92; 95% CI (1.04, 3.52)]. Previous study from Australia [80] have shown a similar association between no physical exercise and LBP. This could be due to the fact that not performing physical exercise might weaken muscles in the lower back and cause a misalignment of the spine, subsequently leading to the development of LBP in teachers [81, 82]. On the other way round, studies done in Tunisia [83], Southern Brazil [84], and China [85] indicated that performing regular physical activity can reduce LBP risk. Likewise, Hosam et al.'s systematic review and meta-analysis of a cross-sectional study corroborated this finding [86]. The possible explanation for this could be that regular physical exercise can enhance muscle strength and endurance, improve cardiovascular function, promote the diffusion of tissue fluid, ensure the absorption of nutrition by bone and muscle tissue, and alleviate muscle fatigue [87]. Therefore, performing regular physical exercise may reduce the risk of LBP [88]. Another reason could be that physical exercise increases blood flow to the spine, which supplies essential nutrients to the structure of the lower back region [89]. This implies that conducting regular physical exercise is imperative to prevent the development of LBP among teachers.

Moreover, the current systematic review and meta-analysis pooled effect size determined that teachers who had sleep problems were two times more likely to develop LBP compared to teachers who did not have sleep problems. This is supported by studies in China [27], India [81], Bangladesh [34], and France [90]. The reasons might be that teachers may have received insufficient rest as a result of insufficient sleep, which aggravated their lower back health issues by increasing their stress levels [81].

Furthermore, empirical evidence from Bangladesh [34], France [91], and elsewhere [74] indicated that a history of previous injury was associated with an increased risk of LBP. Similarly, our study found a significant association between previous injury and LBP in teachers. Congruently, a similar relationship was found between prior injury and back and upper extremity pain in Korean male steelworkers [92]. On top of that, a similar association has been shown among healthcare workers in Italy [93]. The plausible explanation could be that previous injuries weaken the stamina of the back region’s soft tissues, including tendons, cartilage, discs, and nerves, and degrade the natural auto-recovery capability of the musculoskeletal system. This, in turn, reduces the tolerance of the musculoskeletal system for risk factors; thereby easily experiencing LBP. This implies that teachers with a history of previous injury shall be given priority and protected from musculoskeletal disorder risk factors. Though several studies [94–97] found that cigarette smoking was a risk factor for LBP, in this systematic review and meta-analysis, however, no association was found between cigarette smoking and LBP among school teachers in Africa. The findings from a study among Malaysian teachers [98] and a study by Jegnie M et al. [99] mimics similar result.

This systematic review and meta-analysis study suggests that multifaceted intervention strategies targeting socio-demographic, behavioral, physical, and/or educational and ergonomic aspects have been shown to have the greatest improvement in the prevention and management of LBP. Explicitly, risk factors significantly associated with LBP, particularly those identified in this review, should be incorporated into algorithms for the preventive and curative management of LBP. In addition, emphasis should be placed on the implementation and enforcement of the LBP management and control strategy, particularly in developing countries such as Africa to curtail this menace. Above all, future research, especially in Africa, should focus on identifying the barriers to implementation and the effectiveness of ergonomic and other interventions for LBP in school settings, which could help to propose tailored, effective, low-cost, and reasonably simple intervention programs for LBP. A multiple interventions approach to LBP using the results of an intervention trial, such as a cluster randomized controlled trial among teachers and other populations, should also be rigorously implemented in Africa to curb the high levels of LBP.

### Strengths and limitations of the study

This study was a first-of-its-kind systematic review and meta-analysis that estimated the pooled prevalence and associated risk factors of LBP among school teachers in Africa. However, the absence of studies from countries other than the included ones may limit the continental representativeness of the study. The use of only the English language may also limit the conclusiveness of the findings.

## Conclusions and policy implications

In this review, a high pooled prevalence of LBP was found in African school teachers compared to both the global estimate and developed nations. Sex (female), older age, physical inactivity, sleep problems, and a history of previous injury were predictors of LBP. However, no significant pooled effect size was observed between cigarette smoking and LBP among school teachers in Africa. It is recommended that school teachers be provided with and trained on ergonomics, physical exercise, behavioral change, and sleep quality to lessen the impact of LBP. In addition, it is suggested that policymakers and administrators ought to gain awareness of LBP and its risk factors to put existing LBP preventive and control measures into action. Policymakers also need to integrate and strengthen LBP prevention and management strategies into the country's health system management guidelines and implement existing LBP prevention and control measures. Prophylactic management and therapeutic strategies for people with LBP should also be endorsed.

## Supplementary Information

**Additional file 1.** 

## Data Availability

All data generated or analyzed during this study are included in this published article [and its supplementary information files].

## Abbreviations

LBP: Low back pain
MSDs: Musculoskeletal disorders
POR: Pooled odds ratio
GBD: Global burden of disease
YLDs: Years lived with disability
DALYs: Disability-adjusted life years
QOL: Quality of life
PRISMA: Preferred Reporting Items for Systematic Reviews and Meta-Analyses
